# Neonatal Hyperbilirubinemia in a Turkish Cohort: Association of Vitamin B_12_

**DOI:** 10.14740/jocmr2158w

**Published:** 2015-05-08

**Authors:** Nilgun Eroglu, Yasar Kandur, Salih Kalay, Zuhal Kalay, Ozgur Guney

**Affiliations:** a Department of Pediatrics, Necip Fazil City Hospital, Kahramanmaras, Turkey; b Division of Pediatric Nephrology, Necip Fazil City Hospital, Kahramanmaras, Turkey; c Division of Neonatology, Necip Fazil City Hospital, Kahramanmaras, Turkey; d Department of Biochemistry, Necip Fazil City Hospital, Kahramanmaras, Turkey

**Keywords:** Neonatal hyperbilirubinemia, Vitamin B_12_, Hemolysis

## Abstract

**Background:**

Deficiency of vitamin B_12_ (VitB_12_) causes failure of erytrocyte maturation leading to cell lysis. Red blood cell lysis causes excess heme production that ends with hyperbilirubinemia. In this study, we aimed to evaluate the role of VitB_12_ in neonatal hyperbilirubinemia (NNH) with prolonged jaundice and to compare patients with control group who did not develop hyperbilirubinemia.

**Methods:**

A total of 20 patients (M/F = 13/7) with jaundice and 20 healthy controls (M/F = 11/9) were included in the study.

**Results:**

The mean indirect bilirubin level of patient group was 9.91 ± 1.90 mg/dL (6.71 - 15.2 mg/dL) and control group was 3.18 ± 1.24 mg/dL (1.16 - 4.96 mg/dL). The mean VitB_12_ level of patient group was 119.9 ± 43.9 ng/L (42.35 - 178 ng/L) and the control group was 286.17 ± 97.43 ng/L (207.90 - 624.10 ng/L). There was a statistically significant difference in terms of VitB_12_ level (< 0.001) between the study groups.

**Conclusion:**

To our knowledge, this study is the first study showing that low VitB_12_ level has been observed as a risk factor in NNH for the first time in the literature. We suggest that prophylactic use of VitB_12_ by pregnant women so will greatly benefit to prevent VitB_12_ deficiency and its complications in the first years of life such as NNH.

## Introduction

Because of its role in DNA synthesis, vitamin B_12_ (VitB_12_) is essential for cell multiplication during pregnancy [1]. VitB_12_ (stored in liver) is crucial for maturation and proliferation of red blood cells (RBCs). Deficiency of VitB_12_ causes failure of RBC maturation leading to cell lysis [2]. RBC lysis causes excess heme production that ends with hyperbilirubinemia. Since in newborn the functional capacity of liver is only about 1% of the adult liver [3], we supposed that excess bilirubin due to RBC lysis and low functional capacity of liver would result in inefficient clearance of bilirubin so that indirect hyperbilirubinemia develops. In this study, we aimed to evaluate the role of VitB_12_ in neonatal hyperbilirubinemia (NNH) with prolonged jaundice and to compare patients with control group who did not develop hyperbilirubinemia. Prolonged jaundice was defined as total bilirubin level > 5 mg/dL in newborns older than 14 days of age [4, 5].

## Methods

The present study was conducted on a sample of newborns with confirmed diagnosis of prolonged jaundice and a group of sex- and age-matched healthy subjects as controls. Patients group included a sample of newborn with well-established neonatal jaundice, followed up at the Neonatology Clinic, from December 2013 to May 2014, whose parents gave their consent to participate in the study protocol. In order to define the etiology of jaundice, all patients were submitted to a systematic protocol. Briefly, the protocol included ABO group, Rh group, hematocrit, screening for glucose-6-phosphate dehydrogenase deficiency (G6PD), thyroid function tests and urinary culture. Patients who have ABO, Rh incompatibilty, polycythemia, G6PD disease or thyroid disorder were excluded.

### Controls

The control group who had not prolonged jaundice consisted of sex- and age-matched healthy subjects from our Pediatric Clinic. Healthy status was determined through the subjects’ medical history and either a parental report or self-report to rule out the presence of chronic or acute diseases.

### Ethical aspects

The Ethics Committee of School of Medicine of the Sutcu Imam University approved the study.

Venous blood sample was taken from each newborn patient for determination of serum VitB_12_ level. Serum VitB_12_ level was determined by using an autoanalyzer instrument (E170 Roche Hitachi). Serum VitB_12_ was determined by chemiluminescence method.

### Statistical analysis

The Mann-Whitney was used to compare nonparametric continuous variables. The level of significance was set at P < 0.05. All patients informations were anlayzed by SPSS (Statistical Package for Social Science) 16.0 program.

## Results

A total of 20 patients (M/F = 13/7) with jaundice and 20 healthy controls (M/F = 11/9) were included in the study. No differences were observed in general clinical characteristics such as gender, age, birth weight, gestational age and hematocrit levels among patient group and healthy controls (Table 1). The mean age of patient group was 31.95 ± 5.99 days (19 - 39 days) and control group was 33.8 ± 4.14 days (27 - 42 days). The mean indirect bilirubin level of patient group was 9.91 ± 1.90 mg/dL (6.71 - 15.2 mg/dL) and control group was 3.18 ± 1.24 mg/dL (1.16 - 4.96 mg/dL). The mean VitB_12_ level of patient group was 119.9 ± 43.9 ng/L (42.35 - 178 ng/L) and the control group was 286.17 ± 97.43 ng/L (207.90 - 624.10 ng/L). There was a statistically significant difference in terms of VitB_12_ and indirect bilirubin levels (< 0.001) between the study groups.

**Table 1:** Demographic and Clinical Characteristic of Study and Control Group

	Patient group	Control group	P value
Gender (M/F)	13/7	11/9	0.516
Age (day)	31.95 ± 5.99 (19 - 39)	33.8 ± 4.14 (27 - 42)	0.625
Birth weight (g)	3,090 ± 43.67 (2,700 - 3,540)	3,160 ± 282.49 (2,670 - 3,680)	0.350
Gestational age (week)	38.1 ± 0.71 (37 - 39)	38.15 ± 0.74 (37 - 39)	0.815
Serum indirect bilirubin level (mg/dL)	9.91 ± 1.90 (6.71 - 15.2)	3.18 ± 1.24 (1.16 - 4.96)	< 0.001
Hematocrit level (%)	55 ± 8.2	57 ± 9.3	0.450
Serum VitB_12_ level (ng/L)	42.35 - 178 (119.9 ± 43.9)	286.17 ± 97.43 (207.90 - 624.10)	< 0.001

In populations in which the incidence of neonatal jaundice or kernicterus is high, pharmacologic treatment may warrant consideration. However, concerns surround the long-term effects of phenobarbital on these children. Because pharmacologic treatment is probably not justified in populations with a low incidence of severe neonatal jaundice as in our center we did not give phenobarbital treatment [4].

## Discussion

NNH is a frequently encountered problem. Although up to 60% of term newborns have clinical jaundice in the first week of life, few have significant underlying disease [6]. Physiologic jaundice in healthy term newborns follows a typical pattern. All etiologies of jaundice beyond physiologic and breastfeeding or breast milk jaundice are considered pathologic [7]. Pathologic jaundice can result from ABO and Rh incompatibiliy, G6PD deficiency, urinary tract infection, and hypothyroidsm [6]. So that in our study, newborns who have an indirect bilirubin level above 5 mg/dL were submitted to a systemic protocol which composed of ABO, Rh groups, screening for G6PD, thyroid function tests, and urinary culture [5]. Newborns who have none of these risk factors were included in the study.

Here in our study, VitB_12_ level was analyzed for a risk factor of NNH. We found that low VitB_12_ level is a risk factor for developement of NNH. VitB_12_ deficiency is a correctable cause for hemolysis. The mechanism of hemolysis in this condition is ineffective erythropoiesis, where in immature erythrocytes are lysed within the bone marrow itself, resulting in the release of excess quantities of biliverdin, which is ultimately converted to indirect bilirubin [8].

VitB_12_ (cobalamin) is essential for folate metabolism and DNA synthesis, acting as a cofactor for key enzymatic reactions. Deoxyadenosylcobalamin, one of the coenzyme forms of cobalamin, is a cofactor for methionine synthase, the enzyme which converts homocysteine to methionine [9]. In maternal VitB_12_ deficiency, the products homocysteine and methylmalonic acid accumulate and can be transmitted to the fetus. Raised levels of these products have been detected on newborn screening of infants with low VitB_12_ stores [10]. Thus, screening for methylmalonic acid cannot be relied upon to detect infants with low VitB_12_ stores.

VitB_12_ deficiency in newborn, in most cases, results from a maternal deficiency. Maternal causes of infant deficiency can be divided into deficient maternal diet or maternal pernicious anemia in a breastfed infant because infant formulas are supplemented with VitB_12_ [11].

In view of maternal diet serum VitB_12_ concentrations decrease during pregnancy more than can be accounted for by hemodilution [12]. There is some evidence of increased VitB_12_ absorption during pregnancy, with newly absorbed VitB_12_ being more important to placental transport than maternal liver stores [13]. So that, lack of VitB rich diet of the mother may cause VitB_12_ defiency in newborn.

There is a positive relation between maternal and breast-fed infant VitB_12_ deficiency linked to low VitB_12_ levels in breast milk. According to a study by Specker and colleagues, VitB_12_ milk concentrations less than 360 pmol/L, approximately corresponding to a maternal serum VitB_12_ of less than 300 pmol/L, could result in an infant who is biochemically deficient in VitB_12_ [14]. So that low VitB_12_ uptake may cause “not-enough breastmilk-jaundice” that may occur when the baby does not drink enough breast milk. The role of VitB_12_ in breast milk in serum level of newborn shows that a normal maternal blood count is not a reliable marker for deficiency in the infant.

A careful review of available medical literature did yield rare previous reports of severe VitB_12_ deficiency presenting with isolated indirect hyperbilirubinemia without concomitant significant hematologic or neurologic involvement. There are case reports of infant VitB_12_ deficiency being diagnosed on newborn screening. Dasari et al reported a 41-year-old Asian-Indian male who presented with recurrent episodes of jaundice since VitB_12_ deficiency [15].

There are shortcomings in our study. The first one was we do not know the VitB_12_ level of the mothers of the patients and controls during pregancy so that we could not conclude that the relative low levels of VitB_12_ in patients result from the mother’s VitB_12_ status. The second one is that we measured serum VitB_12_ level only once for each patient and number of sample size was relatively small (n = 20) because of financial problems. Briefly, recruitment was conducted to achieve a sample *t*.

However, statistically with minimum sample sizes (n > 20), it may be obvious that the data are drawn from a normally distributed population [16]. So that further studies are needed to collect the samples at least twice in 3-month intervals and with more number of patients for more accuracy.

It has been shown [17] that neonatal jaundice shows statistically significant association with MTHFR 677T (homocysteine metabolism gene) mutation [18] which result elevated homocysteine. Hypomethylation due to MTHFR 677T and folate deficiency, which are independent risk factors could also be a factor leading to predisposition for NNH. So that the role of VitB_12_ in MTHFR 677T mutation related neonatal jaundice need to be functionally established in future prospective studies.

To our knowledge this study is the first study showing that low VitB_12_ level has been observed as a risk factor in NNH for the first time in the literature. However we should mention that NNH, or prolonged jaundice of newborns are multifactorial. Breastfeeding is an important factor as well as genetic background.

We suggest that prophylactic use of VitB_12_ by pregnant women, similar to iron and folic acid prophylaxis, in addition to nutrition education programs and food enrichment, will greatly benefit to prevent VitB_12_ deficiency and its complications in the first years of life such as NNH. This finding in itself emphasizes the need to raise awareness of this presentation among physicians.

## References

[1] Carmel R, Green R, Rosenblatt DS, Watkins D (2003). Update on cobalamin, folate, and homocysteine. Hematology Am Soc Hematol Educ Program.

[2] Khanduri U, Sharma A (2007). Megaloblastic anaemia: prevalence and causative factors. Natl Med J India.

[3] Kawade N, Onishi S (1981). The prenatal and postnatal development of UDP-glucuronyltransferase activity towards bilirubin and the effect of premature birth on this activity in the human liver. Biochem J.

[4] McKiernan PJ (2001). The infant with prolonged jaundice: investigation and management. Curr Ped.

[5] Hannam S, McDonnell M, Rennie JM (2000). Investigation of prolonged neonatal jaundice. Acta Paediatr.

[6] Behrman RE, Kliegman RM, Jenson HB (2000). Jaundice and hyperbilirubinemia in the newborn. Nelson Textbook of pediatrics.

[7] Dennery PA, Seidman DS, Stevenson DK (2001). Neonatal hyperbilirubinemia. N Engl J Med.

[8] Aslinia F, Mazza JJ, Yale SH (2006). Megaloblastic anemia and other causes of macrocytosis. Clin Med Res.

[9] Kapadia CR (1995). Vitamin B12 in health and disease: part I--inherited disorders of function, absorption, and transport. Gastroenterologist.

[10] Campbell CD, Ganesh J, Ficicioglu C (2005). Two newborns with nutritional vitamin B12 deficiency: challenges in newborn screening for vitamin B12 deficiency. Haematologica.

[11] Roumeliotis N, Dix D, Lipson A (2012). Vitamin B(12) deficiency in infants secondary to maternal causes. CMAJ.

[12] Metz J, McGrath K, Bennett M, Hyland K, Bottiglieri T (1995). Biochemical indices of vitamin B12 nutrition in pregnant patients with subnormal serum vitamin B12 levels. Am J Hematol.

[13] Luhby AL, Cooperman JM, Donnenfeld AM, Herrero JM, Teller DN, Wenig JB (1958). Observations on transfer of vitamin B12 from mother to fetus and newborn. American Journal of Disease in Childhood.

[14] Specker BL, Black A, Allen L, Morrow F (1990). Vitamin B-12: low milk concentrations are related to low serum concentrations in vegetarian women and to methylmalonic aciduria in their infants. Am J Clin Nutr.

[15] Dasari S, Naha K, Prabhu M (2012). An unusual cause for recurrent jaundice in an otherwise healthy male. Australas Med J.

[16] Anthony M, Abdul GL (2007). Statistics IV: Interpreting the results of statistical tests. Continuing Education in Anaesthesia. Critical Care & Pain.

[17] Sukla KK, Tiwari PK, Kumar A, Raman R (2013). Low birthweight (LBW) and neonatal hyperbilirubinemia (NNH) in an Indian cohort: association of homocysteine, its metabolic pathway genes and micronutrients as risk factors. PLoS One.

[18] Refsum H, Smith AD, Ueland PM, Nexo E, Clarke R, McPartlin J, Johnston C (2004). Facts and recommendations about total homocysteine determinations: an expert opinion. Clin Chem.

